# PET/CT and SPECT/CT imaging of ^90^Y hepatic radioembolization at therapeutic and diagnostic activity levels: Anthropomorphic phantom study

**DOI:** 10.1371/journal.pone.0271711

**Published:** 2024-02-29

**Authors:** Anna Budzyńska, Agata Kubik, Krzysztof Kacperski, Patrycja Pastusiak, Michał Kuć, Piotr Piasecki, Marcin Konior, Michał Gryziński, Mirosław Dziuk, Edward Iller

**Affiliations:** 1 Department of Nuclear Medicine, Military Institute of Medicine - National Research Institute, Warsaw, Poland; 2 Affidea Mazovian PET-CT Medical Centre, Warsaw, Poland; 3 National Centre for Nuclear Research, Particle Acceleration Physics and Technology Division (TJ1), Otwock—Świerk, Poland; 4 National Centre for Nuclear Research, Radiological Metrology and Biomedical Physics Division (H2), Otwock—Świerk, Poland; 5 Department of Interventional Radiology, Military Institute of Medicine - National Research Institute, Warsaw, Poland; 6 National Centre for Nuclear Research, Radioisotope Centre POLATOM, Otwock - Świerk, Poland; University of Magdeburg, GERMANY

## Abstract

**Purpose:**

Prior to ^90^Y radioembolization procedure, a pretherapy simulation using ^99m^Tc-MAA is performed. Alternatively, a small dosage of ^90^Y microspheres could be used. We aimed to assess the accuracy of lung shunt fraction (LSF) estimation in both high activity ^90^Y posttreatment and pretreatment scans with isotope activity of ~100 MBq, using different imaging techniques. Additionally, we assessed the feasibility of visualising hot and cold hepatic tumours in PET/CT and Bremsstrahlung SPECT/CT images.

**Materials and methods:**

Anthropomorphic phantom including liver (with two spherical tumours) and lung inserts was filled with ^90^Y chloride to simulate an LSF of 9.8%. The total initial activity in the liver was 1451 MBq, including 19.4 MBq in the hot sphere. Nine measurement sessions including PET/CT, SPECT/CT, and planar images were acquired at activities in the whole phantom ranging from 1618 MBq down to 43 MBq. The visibility of the tumours was appraised based on independent observers’ scores. Quantitatively, contrast-to-noise ratio (CNR) was calculated for both spheres in all images.

**Results:**

**LSF estimation**. For high activity in the phantom, PET reconstructions slightly underestimated the LSF; absolute difference was <1.5pp (percent point). For activity <100 MBq, the LSF was overestimated. Both SPECT and planar scintigraphy overestimated the LSF for all activities.

**Lesion visibility**. For SPECT/CT, the cold tumour proved too small to be discernible (CNR <0.5) regardless of the ^90^Y activity in the liver, while hot sphere was visible for activity >200 MBq (CNR>4). For PET/CT, the cold tumour was only visible with the highest ^90^Y activity (CNR>4), whereas the hot one was seen for activity >100 MBq (CNR>5).

**Conclusions:**

PET/CT may accurately estimate the LSF in a ^90^Y posttreatment procedure. However, at low activities of about 100 MBq it seems to provide unreliable estimations. PET imaging provided better visualisation of both hot and cold tumours.

## Introduction

Radioembolization is a method of hepatic tumours treatment where microspheres containing yttrium-90 (^90^Y) are administered into the arterial vasculature of the liver to be delivered into close proximity of the tumour. The tumour is then irradiated by β^−^ particles emitted in ^90^Y decay [1]. The relevant ^90^Y decay data is presented in Table 1.

**Table 1:** Characteristics of radionuclides: Yttrium-90 (^90^Y) and metastable technetium-99 (^99m^Tc) [1–7].

Radionuclide	^90^Y	^99m^Tc
**Physical half-life**	64.041 h	6.0067 h
**Type of decay**	β^-^ emission	isomeric transition
**Daughter product**	zirconium-90 (^90^Zr), stable	technetium-99 (^99^Tc), radioactive (^99^Tc decays with a half-life of 211500 years to the stable ^99^Ru, emitting soft beta radiation without gamma radiation.)
**Additional information**	^90^Y disintegrates by β^-^ emission going directly to the ^90^Zr ground state level with 99.98% probability. The maximum and average energy of the β^-^ particles is 2279.8 keV and 933.6 keV, respectively. The mean tissue penetration is approximately 2.5 mm, with a maximum range of 11 mm. A weak beta branch (0.017%) occurs to the 1760 keV excited level which decays by a E0 gamma transition. This 0(+)-0(+) transition undergoes with the emission of two particles materialized by the emission of two gamma photons, or an electron-positron pair, or internal conversion. The internal pair production branching ratio for the 0(+)-0(+) transition of ^90^Zr was determined to be 32x10^-6^.	^99m^Tc is a nuclear isomer, produced by molybdenum-99 (^99^Mo) /^99m^Tc generator system. Gamma rays produced from isomeric transitions have an energy of 140.511 keV (88.5 photons per 100 disintegrations). There is also a small fraction of photons (0.023 photons per 100 disintegrations) with slightly higher energy of 142.675 keV.

Prior to microsphere radiation therapy, patients undergo relevant planning studies, including mapping angiography and ^99m^Tc-labeled macroaggregated albumin (^99m^Tc-MAA, where ^99m^Tc stands for metastable isotope of technetium-99 and MAA for macroaggregated albumin) imaging [8]. ^99m^Tc characteristics are shown in Table 1. Diagnostic dose of ^99m^Tc-MAA is injected through hepatic arteries, just like ^90^Y microspheres. This pretherapy scout scan is performed to predict ^90^Y microsphere distribution [9]. One of the main goals of the ^99m^Tc-MAA examination concerns the issue of the safety of the planned therapy: estimation of the lung shunt fraction (LSF) as well as detection of potential extrahepatic depositions [10, 11]. High values of lung shunting may be the reason for reducing the therapy dose given (i.e. activity of ^90^Y microspheres), or even a contraindication to radioembolization procedure, if the estimated absorbed dose to the lung is greater than 30 Gy [12–14]. This is because radiation induced pneumonitis and sclerosis due to hepatopulmonary shunting of ^90^Y microspheres is a major toxicity concern in radioembolization procedure [15, 16].

Currently, in many of the nuclear medicine facilities that provide radioembolization treatment, LSF is routinely estimated by ^99m^Tc-MAA planar scintigraphy performed without accounting for attenuation or scatter effects [13, 15, 17, 18]. Because lung and liver have different tissue densities, the LSF will be overestimated when attenuation correction is not applied [15, 19]. Despite the fact that the method based on planar imaging without corrections suffers from quantitative inaccuracy (often resulting in unnecessary treatment modification), it Is consistent with the manufacturers’ recommendations. The use of planar imaging and the 30 Gy limiting dose to the lungs, included in the manufacturers’ instructions, is based on clinical research on radiation pneumonitis, in which the lung-absorbed dose was calculated from ^99m^Tc-MAA planar imaging-based LSFs [9, 15, 20].

In contrast, single photon emission computed tomography combined with computed tomography (SPECT/CT) imaging can significantly improve the accuracy of the LSF assessment [15, 17, 21]. The EANM standard operational procedure [9] recommends that patients with substantial lung shunt visible in planar imaging should have additional SPECT/CT scan to ensure a more accurate quantification of the LSF [9].

Although the ^99m^Tc-MAA imaging usually estimates the distribution of ^90^Y microspheres quite well, several factors may cause a mismatch [9, 22, 23]. The MAA particles and therapeutic microspheres have different physical characteristics: size (the diameter of the microspheres varies from about 15 to 35 μm for glass ones [24] and from 20 to 60 μm for ones made from resin [25], while MAA particles can be from 5 μm to 90 μm in diameter, with the mean of about 15 μm [26, 27], shape, density, as well as the number of particles injected [22, 23, 27]. These differences may lead to variations in their biodistribution. Additionally, the chemical stability of the ^99m^Tc labelled MAA complex is lower than microspheres with encapsulated ^90^Y [13, 18, 28].

Another option that has been proposed is to inject the patient with the so called *scout dose* consisting of a small batch of microspheres, identical to those used for treatment. The goal is to better simulate the treatment [29]. However, the pretreatment activity must be limited due to both the patient radiation protection and the possible distortion of the therapeutic dose distribution by the non-degradable microspheres deposited from the scout dose. For ^90^Y the estimated safety threshold is about 100 MBq [11, 22, 29]. Naturally, such a low activity makes imaging even more challenging.

In this study we aimed to assess the accuracy of LSF estimation by means of phantom imaging, and we analysed both high activity ^90^Y posttreatment scans and pretreatment scans with the ^90^Y activity of ~100 MBq, using different nuclear imaging techniques: hybrid imaging techniques such as PET/CT and Bremsstrahlung SPECT/CT, which are a combination of anatomical and molecular imaging modalities, as well as planar imaging.

In contrast to ^99m^Tc, ^90^Y does not emit any specific nuclear gamma radiation. The imaging is based solely on the Bremsstrahlung generated by the decelerating β^-^ particles. However, the intensity of the Bremsstrahlung per unit activity is low and has a continuous energy spectrum, making it impossible to separate, even partially, the primary and scattered photons. ^90^Y imaging using gamma camera (both planar and SPECT) involves the measurement of Bremsstrahlung photons (primary and scattered) in a wide energy window. Therefore, it poses a major problem when exact attenuation or scatter correction is to be applied [30]. Although dedicated Monte Carlo-based reconstruction algorithms have been developed for which a very good quantitative accuracy of the imaging was reported [31–33], they are still very time consuming and not widely available. An example of a commercially available software package with advanced full Monte Carlo collimator modelling is HybridRecon, launched by Hermes Medical Solutions. It was successfully tested by Porter and al. for post-SIRT ^90^Y Bremsstrahlung SPECT imaging [34]. Still, in most cases, the Bremsstrahlung images are just qualitative (with only approximate attenuation correction) and rather noisy. Additionally, it should be emphasized that planar imaging has its own inherent limitations, when it comes to quantitative analysis [15, 35, 36]. Regarding SPECT, we tested different energy window settings and postprocessing in order to optimise the acquisition protocol for ^90^Y imaging.

For PET, the main fundamental limit to image quality is the very low positron emission probability equal to 32x10^-6^ per decay [4]. The low number of registered true coincidences makes the randoms and background corrections crucial for obtaining quantitatively correct images, and results in relatively large statistical errors.

In the following, the term "high activities" refers to the therapeutic activities of ^90^Y microspheres, i.e. ranging from the maximum value of 1.6 GBq used in this study to approximately 1.0 GBq [37, 38], while the term "low activities" refers to the activities of microspheres lower than 200 MBq, including an activity of ^90^Y of about 100 MBq considered as a scout dose for treatment simulation.

## Materials and methods

Based on our previous experiences with phantom studies [30, 39], we continued the topic of visualization of hot and cold spherical inserts (lesions) in the phantom for different ^90^Y concentrations. Since in those studies the smallest visible cold sphere was 25.4 mm in diameter [30], and in our current work we used higher ^90^Y concentrations, this time we have decided to assess smaller objects. The use of an anthropomorphic phantom (instead of Jaszczak or NEMA phantoms) with liver insert containing fillable spheres allowed us to approximate conditions similar to clinical ones. As in our previous study, tumour visibility analysis based on PET/CT and SPECT/CT imaging data was performed both qualitatively and quantitatively using contrast-to-noise ratio (CNR) as a quantitative parameter [30]. Calculated CNR was then compared to the results of qualitative assessments by human observers. With the clinical aspect of this current work in mind, we also simulated an extrahepatic lesion in order to investigate whether it can be detected using hybrid nuclear imaging techniques, in both ^90^Y post- and pretreatment scans.

### Phantom

An anthropomorphic torso phantom (model ECT/TOR/P) with a cardiac insert (model ECT/CAR/I) was used to simulate a clinical setting. The phantom included a cylindrical spine insert, a fillable liver insert with two fillable spheres (to simulate both a cold no activity and a hot spherical intrahepatic tumour), and lung inserts containing styrofoam beads to simulate lung tissue density. The diameter of the hot sphere was 17 mm, and of the cold one—22 mm. The liver and lungs compartments were filled with ^90^Y chloride to simulate an LSF at the level of 10%. The initial activity in the liver was (1451±22) MBq, including (19.4±1.0) MBq in the hot tumour; the respective activity concentrations were 0.95 MBq/ml (liver) and 7.55 MBq/ml (hot tumour), which meant that the tumour to background ratio was 7.9. The activity in the lungs was (158±8) MBq, and therefore the resulting LSF value was (9.8±0.6)%. The phantom included also an additional cardiac insert with small fillable compartments meant to represent perfusion defects in the myocardium. We have utilised one of those compartments to simulate an extrahepatic deposition of ^90^Y. This volume of 2.6 ml located above the liver and in between the lungs was filled with (9.8±0.5) MBq of ^90^Y chloride. The remainder of the anthropomorphic phantom with the cardiac insert and the cold sphere in the liver were filled with water.

Prior to filling with the radioactive ^90^Y, all compartments of the anthropomorphic phantom were rinsed with a non-radioactive yttrium chloride solution in 0.5 M of hydrochloric acid to prevent adhesion of ^90^Y to the plastic phantom walls.

### Image acquisition and reconstruction

Nine measurement sessions including PET/CT, SPECT/CT and planar imaging were performed over two weeks from August 23^rd^ to September 6^th^, 2021, at activities in the whole phantom ranging from 1618 MBq down to 43 MBq. The last imaging session was performed on the 14^th^ day after phantom filling. Total activities of ^90^Y in the phantom at the beginning of the first scan for each modality (PET, SPECT, and planar imaging) are listed in Table 2. Relative uncertainty of ^90^Y activity for the whole phantom was estimated to be 2%.

**Table 2:** Phantom activities at the time of PET, SPECT, and planar acquisition.

	Days after phantom filling								
	0	1	3	4	7	10	11	12	14
	Total ^90^Y activity in the phantom (MBq)								
PET acquisition	1618	1258	746	578	266	121	94	80	43
SPECT acquisition	1581	1275	766	583	272	127	99	78	43
Planar acquisition	1591	1285	773	592	271	126	95	78	44

### PET/CT imaging

The GE Discovery 710, LSO-based TOF-PET scanner was used for PET/CT imaging. First, a low-dose 64-slice CT scan (preceded by a scout view) was performed to correct the PET emission data for attenuation, and to locate phantom structures for organ delineation. CT scan was acquired with a tube voltage of 140 kV in the helical mode with a current modulation in the range of 40–120 mA. The X-ray tube rotation time was 0.8 s. The helical thickness was 3.75 mm. For the standard type of reconstruction the slice thickness was 1.25 mm. The matrix size was 512x512 [30].

Following CT, three-dimensional PET images were acquired using clinically applied protocol for ^90^Y with acquisition time of 30 min per bed position (15.7 cm with 23% bed overlap). Two bed positions were scanned to fit the entire anthropomorphic phantom in the axial field of view (FOV).

Pet emission data was corrected for geometrical response, detector efficiency, system dead time, random coincidences, scatter and attenuation. Attenuation corrected images were obtained with the use of 3D-OSEM iterative reconstruction method. It was conducted with TOF PET reconstruction algorithm and a resolution recovery algorithm with 4 iterations/32 subsets and a filter cut-off of 3.0 mm. The matrix size was 256x256 [30].

Considering the relatively long acquisition time on PET/CT scanner, compared with typical PET scan times when positron-emitting radiopharmaceuticals such as [^18^F]FDG are used, and low activity in the phantom (especially in the last imaging sessions), a correction for the natural background was applied by performing a series of three PET measurements with the anthropomorphic phantom filled with water containing no activity. The PET/CT data was acquired on different days with the settings described above.

### SPECT/CT imaging

SPECT/CT images were acquired on a hybrid dual-head GE Infinia VCHWK4 gamma camera with HEGP collimators. A single SPECT acquisition was enough to image the entire phantom in the FOV. For each imaging session Bremsstrahlung SPECT was performed three times with different energy window settings, which are shown in Table 3. One of the energy windows, labelled as W3, was divided into 4 narrow emission windows of equal width. This was to provide more accurate attenuation correction based on CT scans. Because different emission energies were acquired in separate sets, a separate CT-based attenuation map was produced for each energy. Then, each set was reconstructed individually and the reconstructed images were summed up.

**Table 3:** Energy window settings for SPECT and planar acquisition.

Energy window label	The width of the energy window	
**W1** ^a^	0–280 keV	
**W2** ^a^	100–200 keV	
**W3**^b^^,^ ^c^	100–200 keV	99.6–124.4 keV
		124.5–149.5 keV
		149.5–174.5 keV
		174.5–199.5 keV

^a^energy window used both for SPECT and planar imaging

^b^energy window used for SPECT imaging only

^c^energy window was divided into 4 narrow emission windows

For each scan 60 projections were acquired in step and shoot mode, with the angular step of 6^O^ in the 360^O^ range, and time per projection of 30 seconds. Body-contour orbit was used to keep the camera detectors close to the phantom. SPECT data was recorded using image matrix 128x128 with a pixel size of 4.42 mm. Image reconstruction was performed using an iterative ordered-subset expectation maximization algorithm (2D OSEM) with 2 iterations and 15 subsets, and the Butterworth filter with a cut-off frequency of 0.5 cycles/cm and a power of 10 was used as a 3D postfilter. No scatter correction or resolution recovery algorithm was included in the reconstruction process. Following emission tomography, a CT scan was performed as follows: the axial mode was used with a tube voltage of 140 kV and a current of 5 mA, and the matrix size was set to 512x512 [30]. Similarly to PET/CT, the CT scan was made for attenuation correction and to support organ delineation [22].

### Planar imaging

Planar scintigraphy was performed by means of static scans with acquisition time of 5 min and the matrix size of 256x256. Two detectors with HEGP collimators were set in H-mode to acquire anterior and posterior images. For each series of measurements, the static images were acquired twice using energy windows of W1 and W2.

### Data analysis

#### Lung shunt fraction estimation

Q. Volumetrix MI application on Xeleris 4.1 XFL was used for organ segmentation and quantification for PET reconstructed images.

For PET imaging, the liver and lung VOI were manually delineated on one CT scan, referred to as “reference scan”, which in turn was rigidly registered to all other CT scans. All VOIs were transformed accordingly from the reference scan to the other CT scans [11, 22]. The LSF was calculated directly as [10, 20, 22]:

$$LSF\;\left[\%\right]=\frac{A_{lung}}{A_{lung}+A_{liver}}\cdot 100\%$$ (1)

where A_lung_ and A_liver_ are the ^90^Y activities in the lungs and liver respectively. Additionally, a volume in the part of the phantom without ^90^Y activity was segmented. This cold region VOI was used as a substitute for lungs with no activity deposited and therefore simulated an LSF = 0% (further referred to as LSF_simulated_). Since the additional volume was smaller than the actual lungs in the phantom, the activity in this cold region was re-scaled to avoid underestimating the LSF_simulated_. The VOI of the cold region was transformed as described above.

To include a correction for the natural background in PET imaging, the mean activity concentration expressed in MBq/ml for the whole phantom volume was determined, and then the background activity both for liver and lung volume was calculated. The LSF_BKG_corrected_ was then calculated according to the formula:

$$LSF_{BGK\_corrected}\;\left[\%\right]=\frac{A_{lung}-K\cdot V_{lung}}{\left(A_{lung}-K\cdot V_{lung}\right)+(A_{liver}-K\cdot V_{liver})}\cdot 100\%$$ (2)

where K is the mean activity concentration determined for the “cold” anthropomorphic phantom, i.e. phantom containing no ^90^Y activity, and V_lung_ (V_liver_) is the volume of the lungs (liver).

SPECT data was reconstructed with the use of Q. Volumetrix MI software on Xeleris 4.1 XFL workstation. However, for organ segmentation and quantification Q.Volumetrix AI available on Xeleris V was used.

To calculate the LSF based on SPECT images the formula Eq (1) was used, with A_lung_ and A_liver_ now being the respective numbers of reconstructed counts in the lungs and the liver [9, 13]. The methods for organ segmentation as well as VOI transformation between different CT scans were similar to those used for PET. The LSF of 0% was simulated in a similar manner as in PET.

Planar data was processed and analyzed on Xeleris 4.1 XFL workstation using basic applications.

For planar imaging, the conjugative view technique was used. A single geometric mean image, composed from the anterior and flipped posterior projection scans, was created each time, and the data analysis was based on geometric mean counts for each ROI [8]. Two-dimensional organ delineation was partially supported by a CT scan acquired from hybrid SPECT/CT acquisition. Both the liver and lung ROI were manually delineated based on additional “thick” coronal views (70.7 mm) of the CT scan. The 2D ROIs created in this way were then copied and positioned in planar images. For each series of measurements, the phantom was not moved between the planar and SPECT/CT imaging.

We used two approaches to calculate the LSF value from planar images. The first was to directly calculate the LSF as defined: the counts from the lung ROI divided by the total counts for the liver and lung ROIs.

The second approach included background correction [9] and required additional background ROIs to be drawn close to the corresponding organ ROIs. The net number of counts in the liver or lungs N_organ_ was then obtained according to the following equation:

$$N_{organ}=N_{organ,\;raw}-\frac{N_{BKG}}{Area_{BKG}}\cdot Area_{organ}\cdot F$$ (3)

where N_organ,raw_ is the number of counts in the organ ROI, N_BKG_ is the number of counts in the associated background ROI, Area (Area_organ_, Area_BKG_) is the size of the ROI. F is a correction factor for background, which was proposed by Buijs et al. [40] as a method to correct for over-subtraction of background activity as a consequence of the volume occupied by the organ. For large organs, like liver or lungs, the diameter of the organ is assumed to be half the thickness of the body, giving an F value of 0.5 [9].

For PET and SPECT images, the uncertainties of LSF values were estimated according to the EANM guidelines [41]. The included sources of errors were the uncertainties of VOI contouring and Poisson statistics of the number of counts in VOIs. For planar images, the appropriate ROIs needed to compute LSF were drawn three times independently by four readers for three cases over the whole range of activities in the phantom. The standard deviations of the obtained LSF values were assumed to represent the measure of uncertainty.

#### Tumour visibility analysis

Qualitative analysis was performed by three observers with at least 2 years of experience in SPECT and PET data analysis. For SPECT, the observers reviewed the images acquired for all the imaging sessions and all energy windows settings resulting in 27 datasets, and for PET imaging, all 9 timepoints were assessed. They were asked to mark the hot and cold tumours in the liver as well as the extrahepatic deposition as either visible or not visible in all of the datasets. Any region that had been marked as discernible from the surrounding background by two or more of the observers was considered visible in the qualitative analysis.

Quantitative analysis was conducted for the hot and cold tumours in the liver. Lesion detection is heavily impacted by both lesion to background contrast and noise in the image, so contrast-to-noise ratio was chosen as a quantifiable parameter of tumour discernibility [30]. The Rose criterion states that an object can be detected in the image if its CNR is over a certain threshold, which is usually between 3 and 5 [7].

As in our previously published work, we used an in-house software to define the lesions based on CT images in their widest cross-section, as well as background regions in close proximity to them. The contours were then transferred onto the nuclear medicine images, and the mean values and standard deviations in the ROIs were computed. CNR calculations were conducted as previously described for NEMA and Jaszczak phantoms [30]. The relevant equations are also provided as supporting information in S3 File.

Due to noise in PET/CT data all of those images were analysed after applying Wiener filter (PSF = 5, noise to signal ratio equal to 0.11).

## Results

### Lung shunt fraction estimation

**Fig 1:**
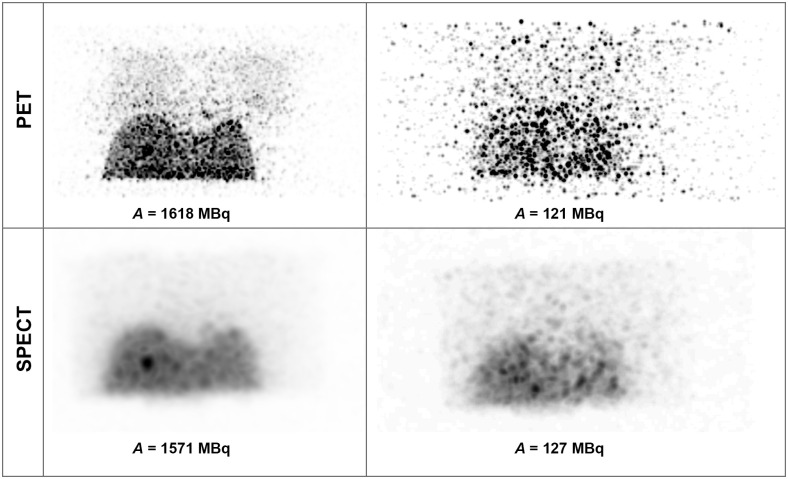
3D MIP PET (top row) and SPECT (bottom row) images of the anthropomorphic phantom for scans performed at high (left) and low (right) activity levels of ^90^Y.

**Fig 2:**
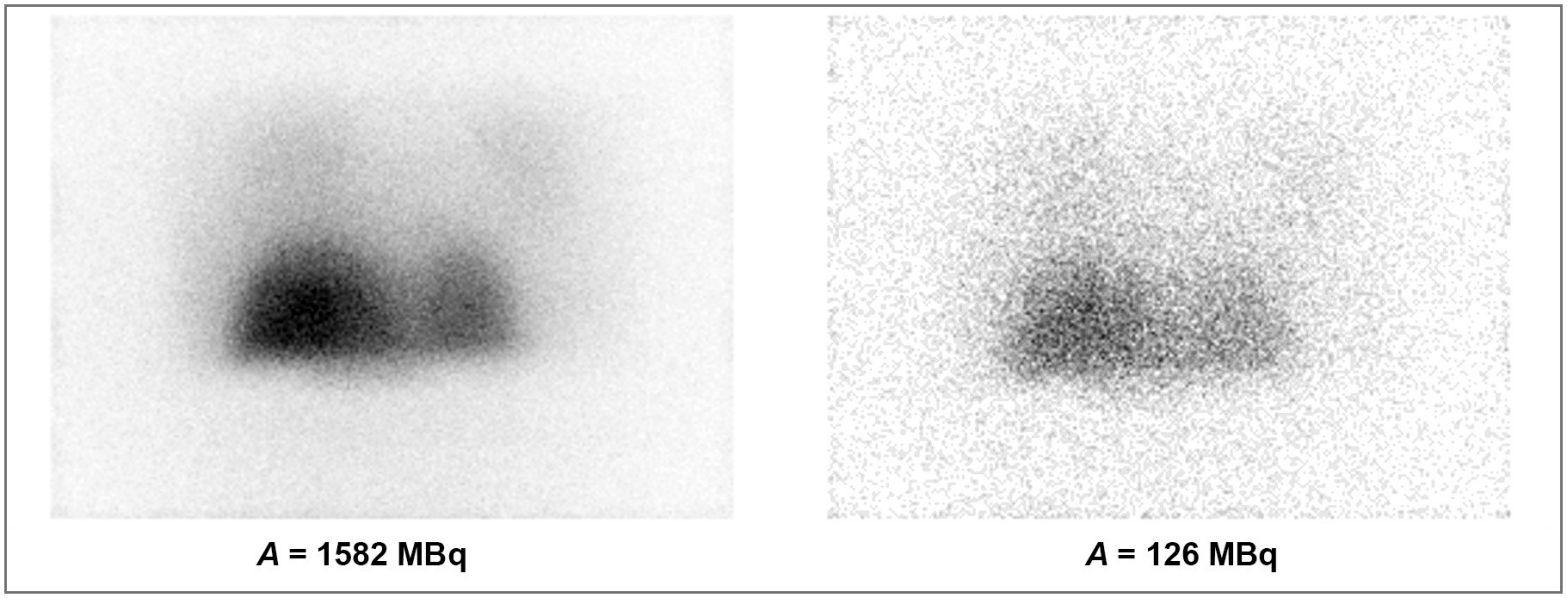
Geometric mean images composed from the anterior and posterior planar images for acquisitions with a total phantom activity of 1582 and 126 MBq.

Fig 1 shows the 3D MIP (Maximum Intensity Projection) of the PET and SPECT images of the anthropomorphic phantom for scans performed at high and low activity levels of ^90^Y, whereas Fig 2 shows the geometric mean images enabling the visual assessment of lung shunt in planar imaging. Both SPECT and planar data presented in Figs 1 and 2 were acquired using energy window of 100–200 keV (W2). PET images acquired at high activity level clearly showed the presence of the lung shunt of a true LSF of around 10%. However, for low activities (below approximately 200 MBq), the lungs and liver were no longer identifiable in PET reconstructions. In SPECT images, the lung shunt was not noticeable, even at a therapeutic activity level of about 1.5 GBq. For planar imaging, one could visually identify the lung shunt only in images obtained with high ^90^Y activities.

**Fig 3:**
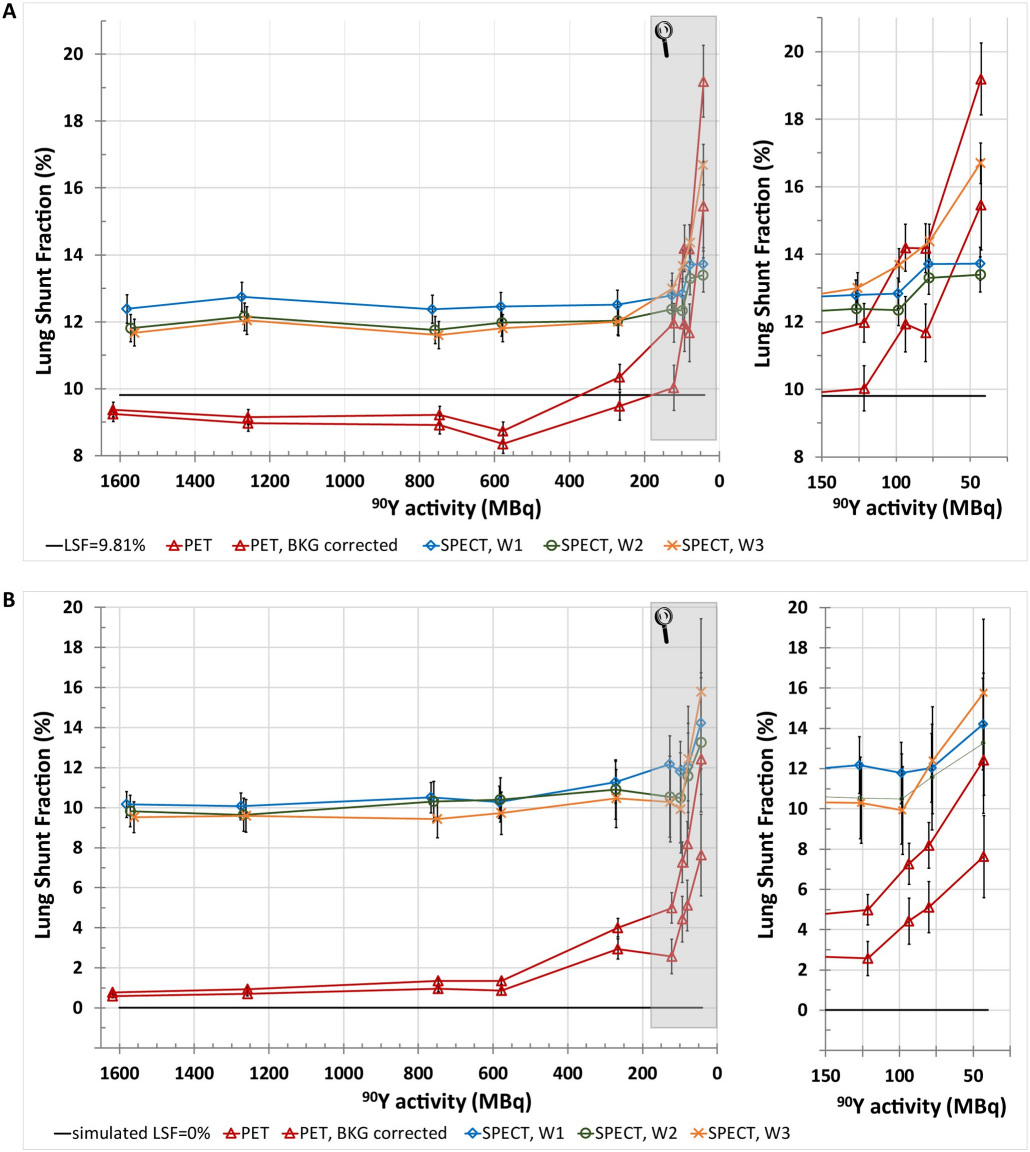
LSF estimated from PET and SPECT imaging for a true LSF of (9.8±0.6)% (A) and LSF_simulated_, i.e. 0% (B) as a function of total ^90^Y activity in the anthropomorphic phantom. For both plots, the highlighted areas are shown in magnification on the right side of the figure. For PET, LSFs computed both with and without the natural background correction are presented. For SPECT modality, the results are shown for acquisitions with different energy window settings: W1, W2, and W3.

**Fig 4:**
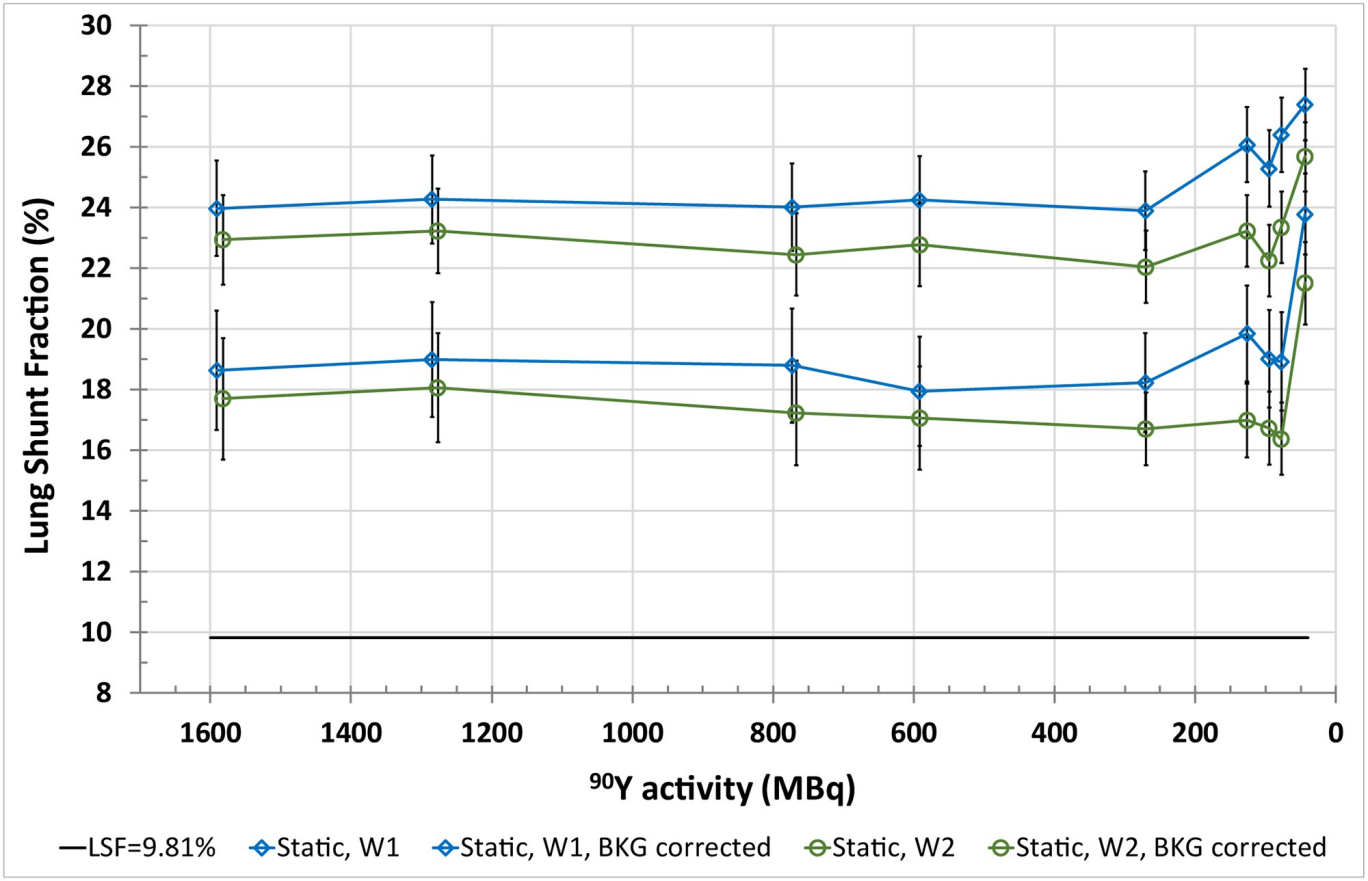
LSF estimated from planar images acquired using energy windows of W1 and W2 for a true LSF of (9.8±0.6)% as a function of total ^90^Y activity in the anthropomorphic phantom. The results are presented for calculations both with and without background correction.

Figs 3 and 4 show the LSF estimated from PET, SPECT and planar imaging as a function of total phantom activity.

#### PET/CT

For PET measurements, the ratios of average ^90^Y activity concentration determined for the whole “hot” anthropomorphic phantom to the natural background activity concentration determined for the “cold” phantom (i.e. phantom containing no ^90^Y activity), ranged from about 100 to 5 on the first and last days of imaging, respectively.

For PET imaging, the LSF was accurately estimated at activities in the phantom ranging from 1618 MBq down to about 200 MBq (the absolute differences between the true value of LSF = 9.8% and the calculated values of LSF were no greater than 1.5 percentage points). However, the LSF estimation was most stable (although slightly underestimated) over the range of high activities down to about 400 MBq. For activities lower than 200 MBq, an increase in the LSF value was observed up to the calculated LSF = (12.0±0.6)% at 120 MBq (overestimating the true value by 2.2 percentage points), although the background correction allowed to reduce it to LSF = (10.0±0.7)% (decreasing the overestimation by a factor of 10). Below 100 MBq, the LSF was greatly overestimated (at 94 MBq the absolute differences between the calculations and the true value were 4.4 percentage points and 2.1 percentage points for PET without and with background correction respectively). These results are presented in Fig 3A.

The LSF_simulated_, based on the VOI with no activity, was stable over the range of high activities down to approximately 500 MBq, and its value was then less than 1% (when the background correction was applied). However, along with the decrease in phantom activity, the calculated background corrected LSF_simulated_ increased to about 4.0% and 7.6% at 100 MBq and 45 MBq, respectively. In the range of low activities the true LSF value of 0% was even more overestimated when assessed using PET without background correction-up to (12.4±1.8)% at 43 MBq. These results are presented in Fig 3B.

#### Bremsstrahlung SPECT/CT

Bremsstrahlung SPECT overestimated the LSF regardless of the energy window setting in the whole range of activities. For activities over 200 MBq, the largest overestimation of the LSF was present for SPECT when the widest energy window was used (W1). Mean LSF values for the seven high activity measurements (where the LSF was approximately constant) were (12.6±0.2)% and (12.1±0.2)% for the W1 and W2 energy windows, respectively. The W3 energy window yielded results very similar to these of W2 in the range of high and medium activities, however, for lower activities, it resulted in the largest overestimation of the LSF (up to nearly 17% at 43 MBq). These results are presented in Fig 3A.

For the region of phantom with no activity (simulated LSF of 0%), the SPECT image-based LSF was greatly overestimated. Mean LSF_simulated_ values from the first seven measurements were: (10.9±0.9)%, (10.3±0.4)%, and (9.9±0.4)% for energy windows of W1, W2, and W3, respectively (Fig 3B).

#### Planar imaging

Planar images greatly overestimated the LSF with differences between the true LSF and the calculated ones up to 17.6 percentage points for W1: true LSF = (9.8±0.6)%, calculated LSF = (27.4±1.2)% and 15.9 percentage points for W2: true LSF = 9.8%, calculated LSF = (25.7±1.2)%, at the lowest activity of about 40 MBq in the phantom. The background correction reduced the LSF overestimation by approximately 5.7 percentage points and 5.5 percentage points for the W1 and W2 energy window, respectively (Fig 4).

### Lesion visibility

**Fig 5:**
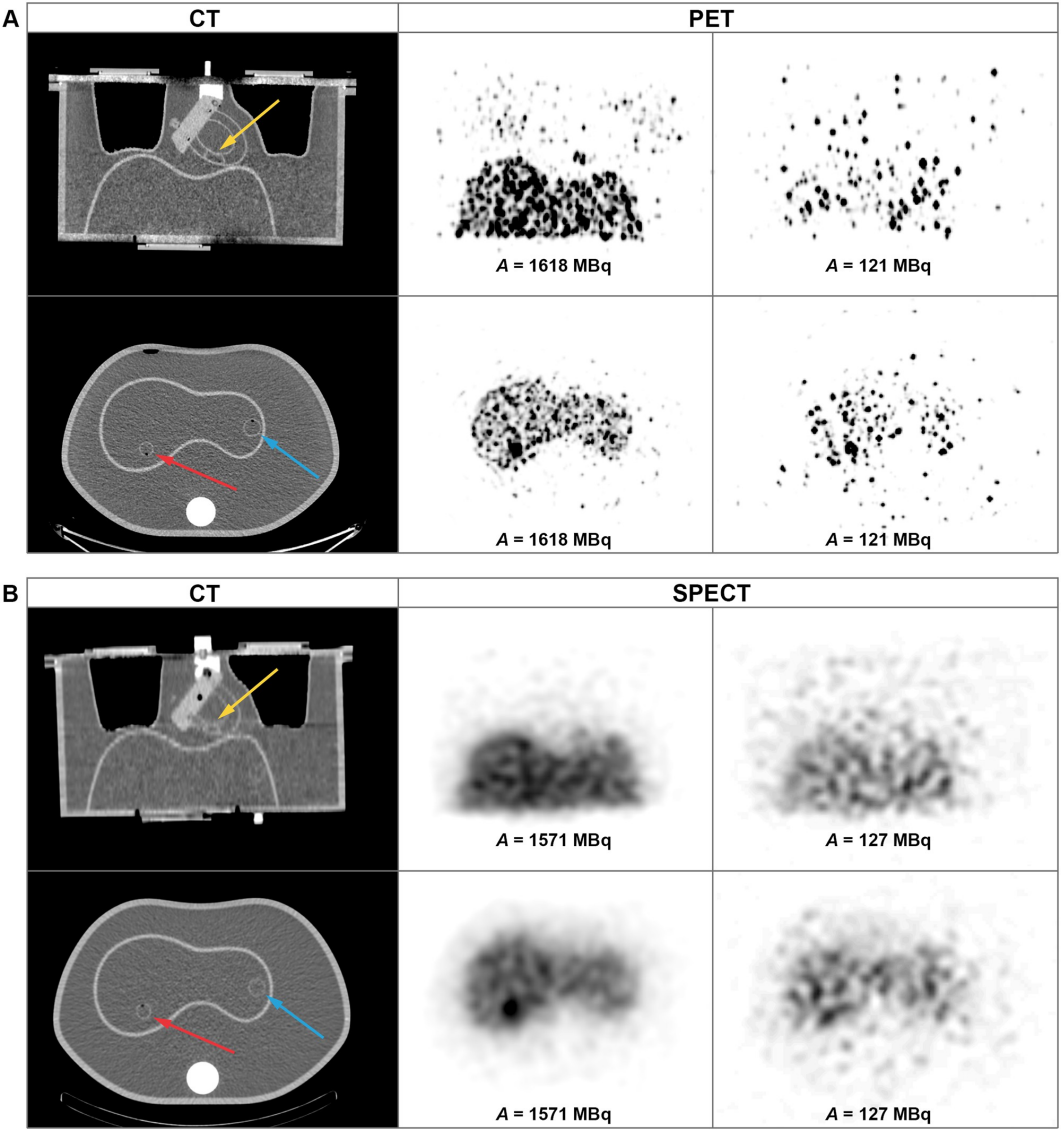
Coronal (top row) and axial (bottom row) views of the PET (A) and SPECT (B) images of the anthropomorphic phantom for scans performed at high and low activity levels of ^90^Y (middle and right column). Left column presents corresponding CT slices. Yellow arrows indicate the location of the extrahepatic deposition while the red and blue ones—the locations of the hot and cold hepatic tumours, respectively.

The reconstructed images (both PET and SPECT) were used for qualitative and quantitative analysis. The observers used both coronal and axial views to determine the visibility of the lesions. They could review single modality as well as fused images. On the other hand, the CNR calculations were performed on the axial views of the phantom (Fig 5). Agreement between the qualitative and quantitative analysis of the tumours’ visibility was deemed good for the assessed modality when for all of the instances in which the lesion was marked as discernible by the observers, its CNR was also greater than 3.

#### Bremsstrahlung SPECT/CT

In the qualitative assessment of SPECT/CT images the hot lesion was visible down to the activity in the whole phantom of 266 MBq (activity in the liver and hot tumour were 239 and 3.2 MBq respectively, with tumour to background ratio of 7.9). However the cold sphere proved not to be visible in any of the acquired images.

**Fig 6:**
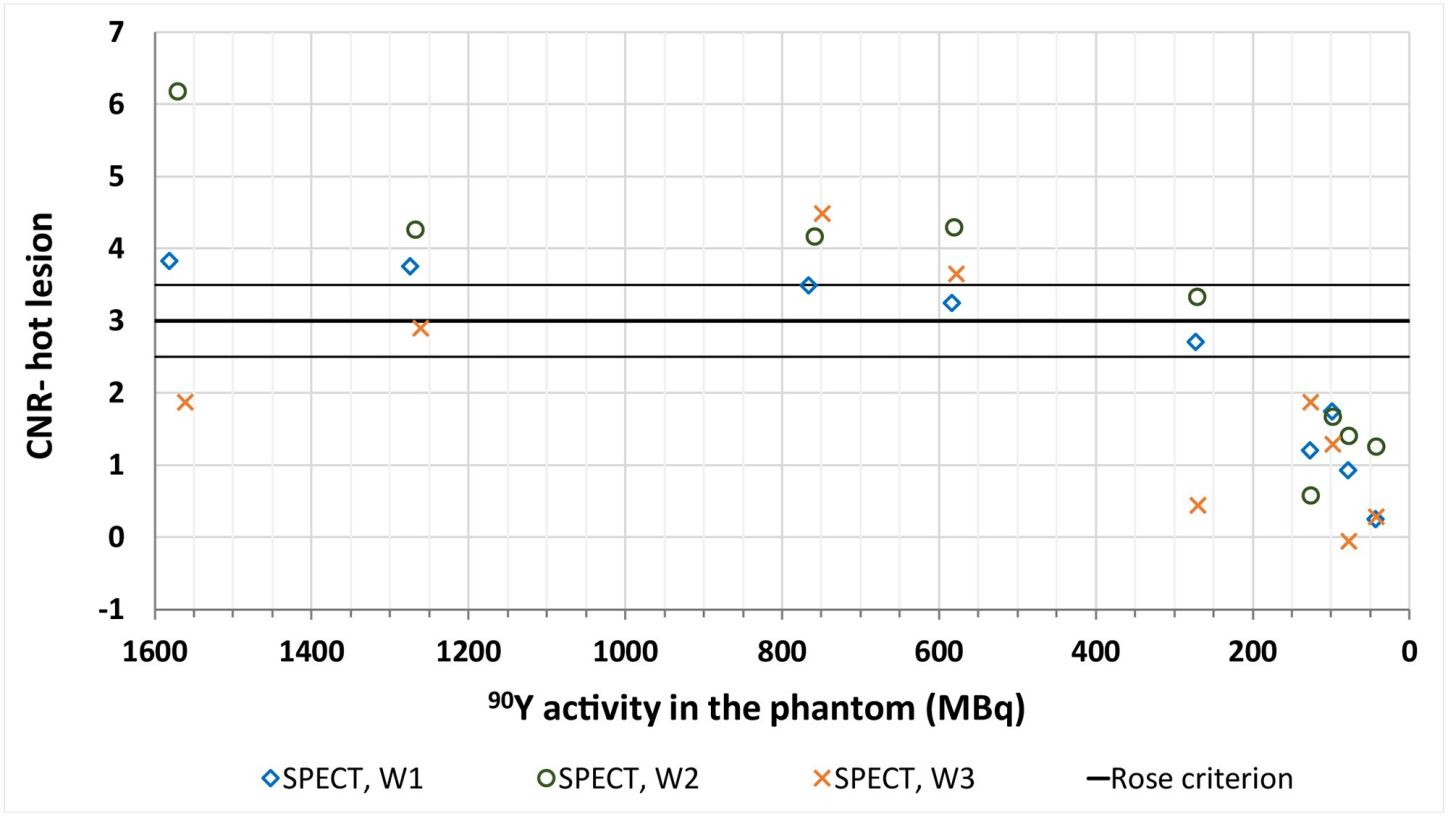
CNR values calculated for the hot lesion in the liver in SPECT/CT imaging for all of the analysed energy window settings. The solid lines represent the border values depending on the Rose criterion (middle line at 3 and supporting ones at 2.5 and 3.5).

**Fig 7:**
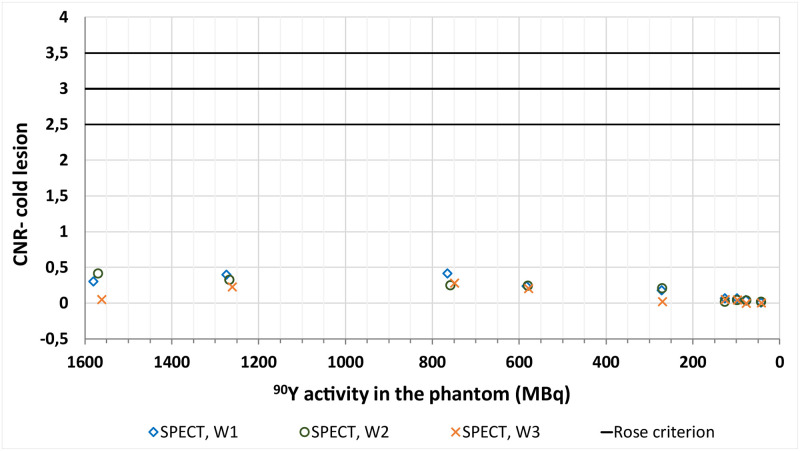
CNR values calculated for the cold lesion in the liver in SPECT/CT for all of the analysed energy window settings. The solid lines represent the border values depending on the Rose criterion (middle line at 3 and supporting ones at 2.5 and 3.5).

The W1 and W2 energy windows acquisitions yielded very good agreement between qualitative and quantitative assessment of tumour visibility: CNR values calculated for all visible lesions were above 3, which is one of the border values suggested by the Rose criterion [7]. For results obtained from the W3 energy window images, the agreement was not satisfactory, as the CNR values of the hot lesion acquired from the first two timepoints were below 3. For the cold lesion, which could not be distinguished in any of the images, the CNR was well below 3, as expected. These results are presented in Figs 6 and 7.

We have also qualitatively analysed the visibility of the extrahepatic concentration (Fig 5). It remained visible up to the 3^rd^ day after phantom filling, when activity in the whole phantom was 747 MBq (in the extrahepatic deposition the activity was then 4.5 MBq). However, it needs to be noted that the last dataset with discernible activity concentration outside of the liver required a careful review of all available cross sections.

#### PET/CT

**Fig 8:**
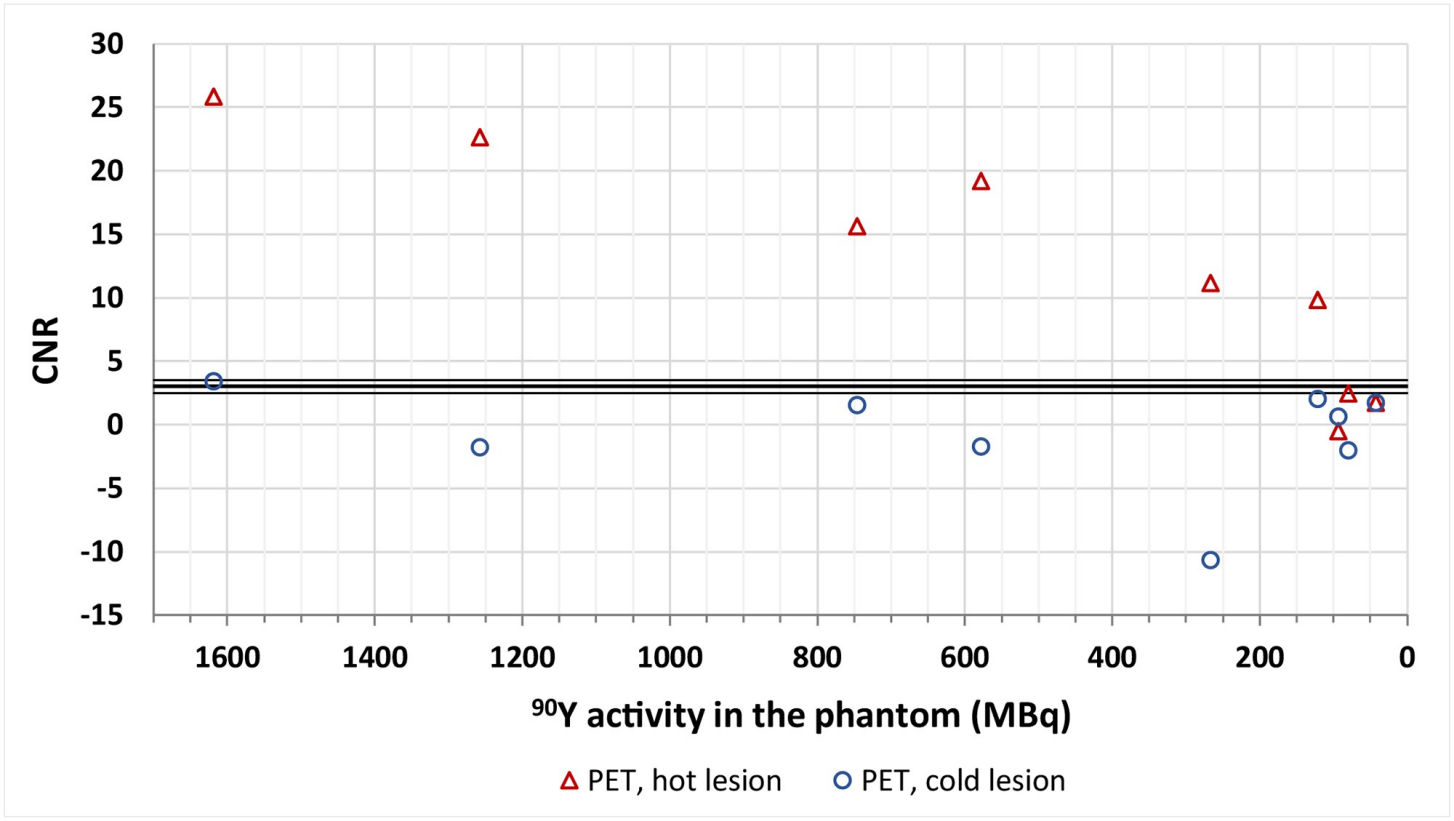
CNR values calculated for the cold and hot lesions in the liver in PET/CT imaging. The solid lines represent the border values depending on the Rose criterion (middle line at 3 and supporting ones at 2.5 and 3.5).

In the qualitative assessment of the PET/CT data the cold sphere was visible in the first acquired dataset, whereas the hot lesion was discernible up to the ninth day after the filling of the phantom (activity in the tumour at this point was 1.46 MBq). These results were in agreement with the calculated CNR values, which for the visible tumours were mostly over the border value of 3 as suggested by the Rose criterion (Fig 8).

As with SPECT, the observers were able to differentiate the extrahepatic lesion up to the 3^rd^ day after phantom filling.

## Discussion

This study aimed at approximating clinical applications not only by using an anthropomorphic phantom (as opposed to NEMA and Jaszczak phantoms), but also by analysing a pretreatment parameter used to qualify patients for therapy with ^90^Y microspheres. In this work we have assessed the accuracy of LSF estimation, the feasibility of imaging hot and cold hepatic tumours as well as extrahepatic lesions using different imaging modalities.

For PET/CT images, we were able to visually distinguish the organs of interest (liver and lungs) for activities in the phantom over 200 MBq. For lower activities, the PET reconstructions are very noisy leading to poor discernibility of the liver, while the lungs cannot be identified at all (Fig 1). On the other hand, in Bremsstrahlung SPECT images (both in MIP and cross-sectional planes) the lung shunt is hardly visible, even for high activities (Figs 1 and 5). The liver is easily identifiable on almost all scans. However, the spill-over effect of activity outside of the liver cannot be ignored, as it adversely affects the possibility of accurate estimation of activity in the background and neighbouring organs. This effect is also connected to lower spatial resolution than in PET technique. Similar spill-over effect could be observed in the planar images. In those images the liver was well distinguishable, however the lungs were identifiable only in images obtained with high activities. It has to be noted, that the visibility of the lungs was worse than in PET reconstructions. Both SPECT and planar imaging was strongly influenced by scattered radiation, due to acquisition of photons in wide energy windows. Neither of those techniques included corrections for this effect, which adversely affects the quality of the images. These qualitative findings are in good agreement with the results of study by Kunnen et al. [22], even though they used a higher LSF value of 15%.

We chose the lung shunt value of 10% because for resin microspheres (SIR-Sphere) it is the experts’ recommended lung shunt threshold beyond which a reduction in isotope activity is strongly advised [1, 42, 43]. Therefore, the LSF of about 10% needs to be detectable and accurately estimated.

The PET data yielded the most accurate LSF estimation, as presented in Fig 3A. In our study the absolute differences of calculated and true LSF values for total activities in the phantom over 200 MBq were less than 1.5 percentage points. In comparison, Kunnen et al. [22] reported differences of less than 2 percentage points for similar activity range. Similarly to the study mentioned above, we have also observed that background correction had the most effect on LSF calculations for low activities, below 200 MBq [22]. Without correction an increasing positive bias of LSF can be observed in this range of activities. This bias is significantly reduced by the background correction for activities about 100–200 MBq, a range which is considered acceptable for pretherapy pilot scans. The bias is still noticeable after background correction, which suggests that further research is needed to find a possibly more accurate correction procedure.

For SPECT data the calculated LSF was between 12% to 13% at total phantom activities over 200 MBq, which meant overestimation of the true LSF = 9.8% by 2.2 and 3.3 percentage points, respectively. Even though this seems like a rather good estimation, it cannot be overlooked, that for the simulated LSF of 0% with cold VOIs the same method yielded LSF values of about 10% (which meant overestimation by 10 percentage points), as shown in Fig 3B. This quantitative result correlates well with the qualitative, visual assessment of the SPECT/CT images, in which the lungs filled with activity did not differ from the cold background. It is worth noting, that using a similar clinical acquisition protocol, Kunnen et al. [22] obtained the LSF values which overestimated the real value of lung shunting by an even greater margin of around 13 percentage points at high activities.

Unlike for SPECT, the PET based calculated LSF for a true LSF of 0% was only slightly overestimated (Fig 3B). This suggests that the obtained results are much more reliable than the values calculated from SPECT data.

In planar imaging we observed a gross overestimation of calculated LSF (Fig 4). The background correction performed according to EANM recommendations [9] allowed for its partial reduction. Nevertheless, the obtained results were definitively worse than those from PET or SPECT. In part, it can be explained by the lack of scatter and attenuation correction, as well as overlapping of different structures.

As a continuation of our previous research, we have analysed the visibility of hot and cold tumours in the liver. We have found that PET acquisitions provided better images in terms of lesion detectability. The hot lesion was visible even for low activities (i.e. 121 MBq, images in the right column in Fig 5A), while the cold lesion was reported to be visible both qualitatively and quantitatively only for the highest activity of ^90^Y (i.e. 1618 MBq, the middle column in Fig 5A).

The cold tumour was not visible in any of the acquired SPECT scans (Fig 5B). This might be explained by poor spatial resolution and spill-over activity from the liver. The acquired CNR values are consistent with the lesion’s discernibility and our previous findings.

We have found good correlation between CNR and hot tumour visibility for energy windows W1 and W2, with higher values calculated for the latter. For the W3 energy window there were significant discrepancies. The first two acquisitions, which should have provided the highest CNR values, proved to be below the Rose criterion border value of 3. It might have been influenced by the fact that the images analysed for the W3 energy window session were summed reconstructions, as opposed to W1 and W2 sessions. The choice of energy window seems to have rather minor effect on both the detectability of lesions and the accuracy of LSF estimation.

Resolution recovery, which is based on modelling the geometric response of the collimator to a point source, is intended to improve spatial resolution by correction of collimator-detector blurring [44]. However, for the Bremsstrahlung radiation generated by the ^90^Y point response function is much broader and in fact should be a part of proper scatter modelling, which was not available on our gamma camera. In this case, it is better not to use collimator modelling at all, as it would be inherently incorrect.

For SPECT imaging based on registration of Bremsstrahlung, the reconstructions commonly used in clinical practice suffer inherently from insufficient attenuation correction and lack of scatter correction. Dewaraja et al. [32] in their work on Monte Carlo scatter modelling for accurate ^90^Y bremsstrahlung SPECT/CT imaging showed that reconstruction without scatter correction significantly reduces the accuracy of quantitative measurements resulting in underestimation of intrahepatic lesion activity and overestimation of the normal liver activity, as well as large overestimation of ^90^Y activity in the lungs. Apart from affecting quantitative analysis, the lack of scatter correction has adverse effects on image quality. For example, it causes the deterioration of the lesion-to-normal-liver contrast [32, 45]. Therefore, any calculations based on the images affected by those problems cannot be considered truly quantitative, and have to be interpreted cautiously as estimates only. These shortcomings might be overcome by implementation of Monte Carlo based reconstruction algorithms [32, 46]. It has been shown that incorporating the full Monte Carlo collimator simulation (in addition to considering scatter within the object) into OSEM iterative reconstruction significantly increases contrast recovery (without degrading image quality) and thus can improve the accuracy of image quantification [34]. However, Monte Carlo based reconstruction methods are time and labour consuming and are still not easily accessible for many smaller nuclear medicine centres. On the other hand, a more elaborate background correction method could improve the quantitative accuracy of the estimated LSF.

Our study suggests that PET/CT provides better images for high activity posttreatment imaging of patients undergoing therapy with ^90^Y microspheres than Bremsstrahlung based SPECT/CT. Lowering the activity to levels acceptable in pretreatment scans (about 100 MBq), poses a great challenge in imaging. Its main goal is to reliably assess the LSF. Our work demonstrates that PET/CT yields the best results among the tested methods. However, it seems that it may have some inherent limitations in imaging (low counts in the image), which explains the difficulties with image interpretation and analysis at the lowest activities. SPECT/CT could be considered for this purpose, if suitable corrections can be applied. As for planar imaging, it did not prove to provide enough information for it to be clinically useful for pretreatment ^90^Y scans.

## Supporting information

S1 File PET, SPECT and planar data for LSF calculations. (XLSX)

S2 File CNR for SPECT and PET. (XLSX)

S3 File CNR calculation methods. (DOCX)
