# Supplementary material for: PET/CT and SPECT/CT imaging of 90Y hepatic radioembolization at therapeutic and diagnostic activity levels: Anthropomorphic phantom study
Source: PLoS One. 2024 Feb 29;19(2):e0271711. doi: 10.1371/journal.pone.0271711 (PMC10903856; doi:10.1371/journal.pone.0271711)
Supplement: S3 File — (DOCX) [file pone.0271711.s003.docx]

In all analysed images we have defined the cold and hot foci based on CT images. All regions of interest (ROIs) where then transferred onto the corresponding SPECT and PET data in order to calculate the quantitative parameters.

For the cold lesion in the in SPECT data we have used the following equations for our calculations (1):

| $CNR= \frac{C}{RMSN}=\frac{\frac{S_{b}-S_{mROI}}{S_{b}} \cdot100\%}{\frac{\sigma_{b}}{S_{b}} \cdot100\%}=\frac{S_{b}-S_{mROI}}{\sigma_{b}}$ | (1) |
| --- | --- |

where C is the contrast of the cold sphere, S_b_ - mean background signal, S_mROI_ - minimal signal in the analysed ROI, RMSN - root mean square noise and σ_b_ – standard deviation in the background.

For the PET images of the cold focus we have implemented the following calculation methods (2):

| $CNR= C_{ROI} \cdot\sqrt{n_{ROI}} \cdot\frac{S_{b}}{\sigma_{b}}=\frac{S_{ROI}-S_{b}}{S_{b}} \cdot\sqrt{n_{ROI}} \cdot\frac{S_{b}}{\sigma_{b}}=\frac{S_{ROI}-S_{b}}{\sigma_{b}}\cdot\sqrt{n_{ROI}}$ | (2) |
| --- | --- |

where C_ROI_ is the sphere to background contrast, n_ROI_- the number of pixels in the ROI, S_ROI_- the mean signal in the ROI.

For hot spheres we have modified the equation for contrast to (3):

| $C= \frac{S_{ROI}-S_{b}}{S_{ROI}} \cdot100\%$ | (3) |
| --- | --- |

Where S_ROI_ is the mean signal in the hot sphere. The RMSN was used as the measure of noise in the data.

Due to noise in the PET images all calculations were conducted after applying the Wiener filter (PSF = 5, noise to signal ratio = 0.11) (25).

References

[1] Graham LS, Fahey FH, Madsen MT, Van Aswegen A, Yester M V. Quantitation of SPECT Performance: Report of Task Group 4, Nuclear Medicine Committee. Med Phys. 1995;22(4):401–9.

[2] Bao Q, Chatziioannou AF. Estimation of the minimum detectable activity of preclinical PET imaging systems with an analytical method. Med Phys. 2010;37(11):6070–83.

[3] Cherry SR, Sorenson JA, Phelps ME. Image Quality in Nuclear Medicine. In: Physics in Nuclear Medicine. 4th ed. Elsevier; 2012. p. 239–43.
